# Differential activation of placental unfolded protein response pathways implies heterogeneity in causation of early- and late-onset pre-eclampsia

**DOI:** 10.1002/path.4394

**Published:** 2014-08-06

**Authors:** Hong Wa Yung, Daniel Atkinson, Tim Campion-Smith, Matts Olovsson, D Stephen Charnock-Jones, Graham J Burton

**Affiliations:** 1 Centre for Trophoblast Research, University of CambridgeUK; 2 Department of Women's and Children's Health, Uppsala UniversitySweden; 3 Department of Obstetrics and Gynaecology, University of CambridgeUK; 4 National Institute for Health Research, Cambridge Comprehensive Biomedical Research CentreUK

**Keywords:** placenta, pre-eclampsia, pregnancy, growth restriction, cell stress responses, unfolded protein response

## Abstract

Based on gestational age at diagnosis and/or delivery, pre-eclampsia (PE) is commonly divided into early-onset (<34 weeks) and late-onset (≥34 weeks) forms. Recently, the distinction between ‘placental’ and ‘maternal’ causation has been proposed, with ‘placental’ cases being more frequently associated with early-onset and intrauterine growth restriction. To test whether molecular placental pathology varies according to clinical presentation, we investigated stress-signalling pathways, including unfolded protein response (UPR) pathways, MAPK stress pathways, heat-shock proteins and AMPK*α* in placentae delivered by caesarean section for clinical indications at different gestational ages. Controls included second-trimester, pre-term and normal-term placentae. BeWo cells were used to investigate how these pathways react to different severities of hypoxia–reoxygenation (H/R) and pro-inflammatory cytokines. Activation of placental UPR and stress-response pathways, including P-IRE1*α*, ATF6, XBP-1, GRP78 and GRP94, P-p38/p38 and HSP70, was higher in early-onset PE than in both late-onset PE and normotensive controls (NTCs), with a clear inflection around 34 weeks. Placentae from ≥ 34 weeks PE and NTC were indistinguishable. Levels of UPR signalling were similar between second-trimester and term controls, but were significantly higher in pre-term ‘controls’ delivered vaginally for chorioamnionitis and other conditions. Severe H/R (1/20% O_2_) induced equivalent activation of UPR pathways, including P-eIF2*α*, ATF6, P-IRE1*α*, GRP78 and GRP94, in BeWo cells. By contrast, the pro-inflammatory cytokines TNF*α* and IL-1*β* induced only mild activation of P-eIF2*α* and GRP78. AKT, a central regulator of cell proliferation, was reduced in the < 34 weeks PE placentae and severe H/R-treated cells, but not in other conditions. These findings provide the first molecular evidence that placental stress may contribute to the pathophysiology of early-onset pre-eclampsia, whereas that is unlikely to be the case in the late-onset form of the syndrome. © 2014 The Authors. *The Journal of Pathology* published by John Wiley & Sons Ltd on behalf of Pathological Society of Great Britain and Ireland.

## Introduction

Pre-eclampsia (PE) affects 3–8% of pregnancies and is associated with the development of *de novo* hypertension, proteinuria, liver dysfunction and other systemic disturbances after 20 weeks of gestation 1. The aetiology of the syndrome remains elusive, although it is widely recognized that the placenta is a necessary and sufficient cause. However, major differences in the clinical presentation indicate heterogeneity in the underlying pathophysiology 2,3. Attempts have therefore been made to classify the syndrome into distinct subgroups 4,5. Several schemes have been proposed, and the most common is to divide cases clinically into early- and late-onset, according to the gestational age at diagnosis or when delivery is necessitated 4. The majority (75%) of cases are late-onset, usually defined as the occurrence after 34 weeks gestational age. Of the two forms, early-onset is often considered the more severe, as it is associated with a higher rate of intrauterine growth restriction (IUGR) and risk of maternal cardiovascular complications after delivery 6,7. Moreover, there is a greater prevalence of placental lesions indicative of maternal malperfusion 8–10, as confirmed by a recent magnetic resonance imaging study 11.

In view of these differences, it has been proposed that PE can be classified on the basis of the pathophysiology into ‘placental’ and ‘maternal’ causation 12. In the former, it is postulated that malperfusion leads to placental stress and the release of cytokines and angiogenic regulators that cause maternal endothelial cell activation. In the latter, it is believed that the same final stage is reached due to a predisposing exaggerated maternal endothelial sensitivity to factors emanating from a relatively normal or excessively large placenta.

A major tenet on which this latter classification is based is that normal pregnancy is a pro-inflammatory state, with activation of circulating immune cells 13 and elevated systemic oxidative stress as gestation advances 14. Therefore, ‘maternal’ PE may simply be the result of an excessive maternal response towards existing inflammation 15. This argument is supported by accumulating evidence that women suffering from chronic systemic inflammation, due to conditions such as renal disease, autoimmune diseases or metabolic syndromes, are at higher risk of developing PE towards term 16. Furthermore, there is little evidence of abnormal uterine perfusion or placental pathology in cases presenting towards term 17–19. By contrast, in ‘placental’ PE it is widely accepted that there is deficient remodelling of the maternal spiral arteries 17, as indicated by abnormal uterine artery Doppler waveforms. Deficient remodelling is predicted to result in high-velocity, intermittent, maternal blood flow within the inter-villous space 20, which can induce placental oxidative stress through high shear rates and an ischaemia/hypoxia–reperfusion type of injury 21,22. Placental oxidative stress causes release of anti-angiogenic factors into the maternal circulation 23 and levels of anti-angiogenic factors, such as sFlt1, in the maternal circulation during the second trimester of pregnancy are strongly positively correlated with the time of onset of the syndrome 24.

Ischaemia/hypoxia–reperfusion is a strong inducer for oxidative stress, and also activates endoplasmic reticulum (ER) stress 25,26. The ER is the site of biosynthesis of polypeptide hormones, growth factors and plasma membrane proteins and their post-translational modification. Genetic manipulation of ER stress causes placental insufficiency, which in turn restricts fetal development and growth 27. Accumulation of unfolded and misfolded proteins activates ER stress-response pathways, also referred to as the unfolded protein response (UPR). The UPR comprises three highly conserved signalling pathways: the PERK–eIF2*α* pathway, which attenuates non-essential protein synthesis; and the ATF6 and IRE1–XBP1 pathways, which increase folding capacity by up-regulation of the ER chaperones GRP78 and GRP94 and phospholipid biosynthesis. In addition, the endoplasmic reticulum-associated protein degradation (ERAD) pathway facilitates protein degradation 28. We have recently demonstrated activation of ER stress pathways in placentae from cases of IUGR, with greater activation in those complicated with pre-eclampsia (IUGR + PE) 26.

In this study, we tested whether molecular evidence of placental stress responses supports the concept that causation of early-onset PE is indeed related to placental pathology, while late-onset PE is predominantly associated with other factors, such as maternal metabolic syndromes or endothelial sensitivity. Following our previous finding of high ER stress in IUGR + PE placentae, we examined activation levels of UPR pathways and other common stress signalling pathways, including AMPK*α*, p38–JNK stress signalling pathways and cytosolic heat shock proteins (HSPs) in placentae delivered from pre-eclamptic patients at gestational ages of 24–39 weeks. To determine the stimulus required to generate equivalent changes *in vitro*, we exposed human choriocarcinoma cells (BeWo) to different severities of hypoxia–reoxygenation or treatment with pro-inflammatory cytokines.

## Materials and methods

Details of chemicals and reagents used are described in Supplementary information on materials (see supplementary material).

### Study population and placental sample collection

All placental samples were obtained with local ethical permission and the patients' informed written consent. The detailed criteria for recruitment of patients for this study have been described previously 3. Briefly, pre-eclampsia was defined as new-onset hypertension (≥140/90 mmHg) observed on at least two separate occasions, 6 h or more apart, combined with proteinuria (a 24 h urine sample showing ≥ 300 mg/24 h). The control group was from healthy normotensive term patients that displayed no abnormalities on routine scans. Women with essential hypertension, diabetes mellitus or pre-existing renal disease were excluded.

Both PE and term control placentae were obtained from elective non-laboured caesarean deliveries. Samples of second-trimester placentae were obtained using an ultrasound-guided chorionic villus sampling technique prior to termination of pregnancy for psychosocial reasons. Pre-term controls were from pregnancies complicated with a variety of obstetric conditions, including acute chorioamnionitis, placental fragmentation, subchorionic and intraparenchymal haemorrhage. These placentae were delivered vaginally, after spontaneous labour. For each placenta, four to six small pieces of tissue from separate lobules were rinsed three times in saline, blotted dry and snap-frozen in liquid nitrogen within 10 min of delivery; the samples were stored at –80 °C.

### Cell culture

Villous trophoblast-like BeWo cells were used as primary cultures of trophoblast do not proliferate *in vitro*. BeWo cells are traditionally cultured in Dulbecco's modified Eagle's medium (DMEM)/F12 containing 17.1 mm glucose. In hypoxia–reoxygenation experiments the high glucose concentration leads to severe acidosis and likely contributes to the global protein synthesis inhibition observed (data not shown). Therefore, BeWo cells were adapted to growth in the more physiologically appropriate glucose concentration of 5.5 mm (in DMEM/F12). Initially the cells grew more slowly, but after 10 passages the cell proliferation rates were indistinguishable. All the experiments described were carried on BeWo cells after at least 20 passages in 5.5 mm glucose. The protocol for culturing BeWo cells has been described previously 29.

### Different severities of hypoxia–reoxygenation incubation

To mimic different degrees of oxidative cellular stress, an *in vitro* model using cycles of different oxygen levels was developed. For the mild and severe oxidative insults, cells were incubated in serum-free medium (DMEM/F12, 5.5 mm glucose) under repetitive 6 h cycles of 5% and 20% O_2_ (5/20 H/R) or 1% and 20% O_2_ (1/20 H/R), respectively (ExVivo, BioSpherix, USA) for 24 h. Constant 20% O_2_ was used as a normoxic control. The syncytiotrophoblast layer is sensitive to oxidative damage *in vivo*, and so BeWo syncytialization was induced prior to study (forskolin A, 10 µm for 24 h). Syncytialization was confirmed by immunocytochemical staining for desmoplakin, and the cultures showed multinucleated cells and increased E-cadherin (data not shown).

Syncytialized BeWo cells do not proliferate; therefore, non-syncytialized BeWo cells were used to investigate the effects of different severity of H/R on cell proliferation. Briefly, cells were seeded at low density, cultured in the presence of 10% serum for 48 h under different H/R conditions and proliferation measured as previously described 26.

### Electron microscopy

Details are described in a previous study 29.

### Western blotting

Details are described in a previous study 29.

### Statistical analysis

Differences were tested using non-parametric Kruskal–Wallis test with Dunn's multiple comparison test, with *p ≤* 0.05 considered significant. Correlations with gestational age at delivery were tested using the Pearson correlation, with *p ≤* 0.05 considered significant. Power regression lines were fitted to display the relationship. For cell culture experiments, the paired non-parametric Friedman test with Dunn's multiple comparison test or paired non-parametric Wilcoxon test were used, with *p ≤* 0.05 considered significant. All statistical analysis was performed using GraphPad Prism v 6.0. Cluster analysis using Ward's agglomerative method was carried out using the statistical language R (v 3.0.1).

## Results

Twenty-four placental samples from pre-eclamptic pregnancies of gestational age in the range 24–39 weeks at the time of delivery, eight normotensive term control (NTC) and seven pre-term ‘control’ (PTC) placentae were used for this study. Their clinical features are presented in Table1 (see also supplementary material, Table S1). Additionally, samples from second-trimester (17.3 ± 0.5 weeks, *n =* 7) placentae were included to investigate the effects of gestational age.

**Table 1:** Clinical parameters recorded for the 24 pre-eclamptic, eight normotensive term control (NTC) and seven preterm 'control' (PTC) placental samples used in the study

	<34 weeks PE (*n* = 15)	>34 weeks PE (*n* = 9)	Term control (*n* = 8)	*Pre-term control (*n* = 7)	*p* (<0.05)
Parameter	1	2	3	4	
Gestational age (weeks)	31.9 ± 3.1	37.2 ± 1.7	39 ± 0.8	29 ± 2.9	1 vs 2; 1 vs 3; 2 vs 4; 3 vs 4;
Maternal age (years)	31.1 ± 4.4	32.6 ± 5.3	32.8 ± 6.2	29.1 ± 6	n.s.
BMI	27.3 ± 5.1	26.1 ± 7.3	24.5 ± 4.7	20.5 ± 8	1 vs 4
BP systolic	164.6 ± 16.2	146 ± 11.8	118.3 ± 8.4	112.9 ± 11	1 vs 3; 1 vs 4
BP diastolic	99.6 ± 6.6	95.9 ± 11.6	73.6 ± 11.4	72.3 ± 11.4	1 vs 3; 1 vs 4; 2 vs 3; 2 vs 4
Fetal weight (g)	992 ± 345	2828 ± 741	3960 ± 651	1430 ± 670	1 vs 2; 1 vs 3; 3 vs 4
Placental weight (g)	260 ± 119	605 ± 331	679 ± 69	257 ± 66	1 vs 2; 1 vs 3; 2 vs 4; 3 vs 4

The data are presented as mean ± SD; however, non-parametric Kruskal–Wallis test with Dunn's multiple comparison test was used to describe statistical significances among groups, with *p ≤* 0.05.

^*^Placental tissues were collected from vaginal deliveries and were complicated by conditions such as placental fragmentation, subchorionic and intraparenchymal haemorrhage and acute chorioamnionitis.

vs, versus.

### Activation of UPR pathways in early-onset, but not in late-onset PE and normotensive control placentae

**Figure 1:**
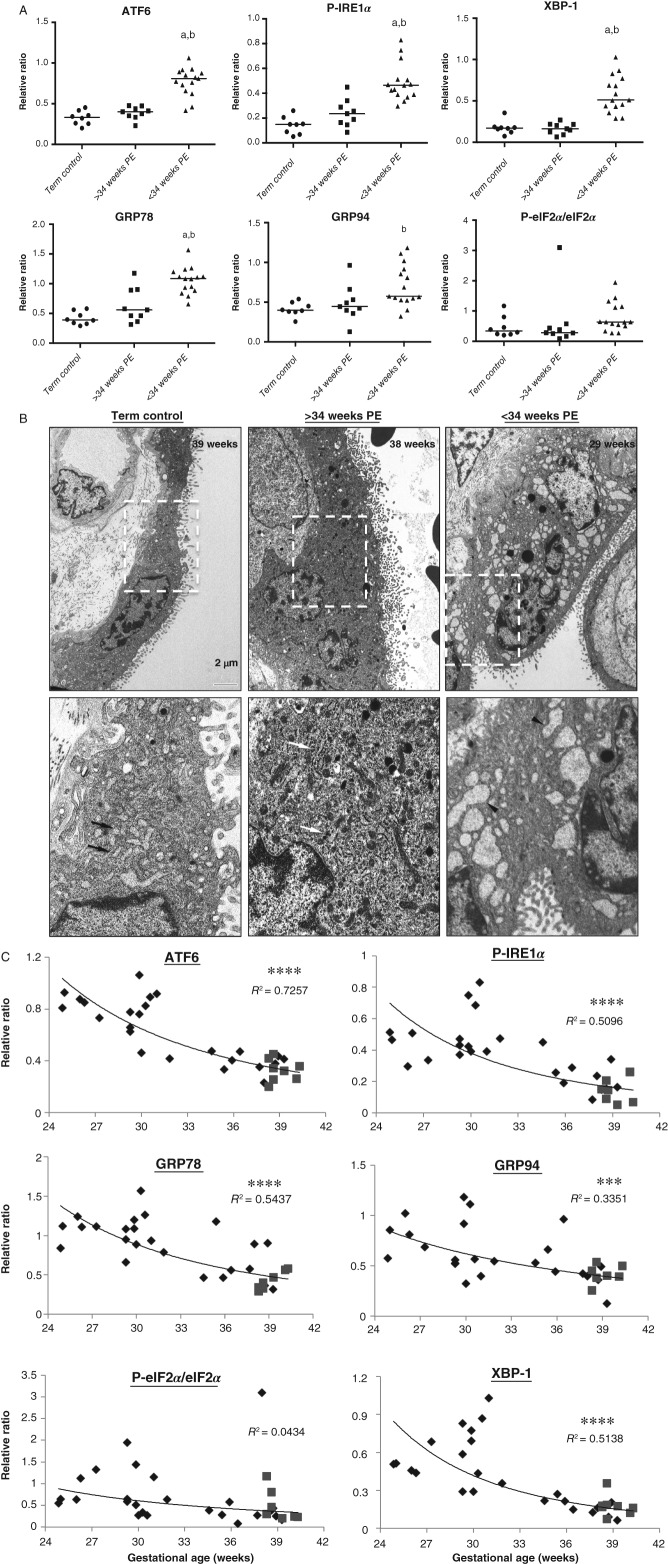
Continued. High activation of UPR pathways in early-PE (<34 weeks) but not late-PE (≥34 weeks) placentae and a strong negative correlation between activation and gestational age of PE placentae. (A) Comparison among early-PEs, late-PEs and NTCs; the intensity of bands was quantified and normalized with *β*-actin: data are presented as medians; a non-parametric Kruskal–Wallis test with Dunn's multiple comparison test was used, with *p ≤* 0.05 considered significant; a, b, significant differences between < 34 weeks PEs and term controls or ≥ 34 weeks PEs, respectively; term con, term controls. (B) Electron micrographs show dilation of ER cisternae in the early-PE placenta (29 weeks) but not in the late-PE (38 weeks) and NTC (39 weeks) placentae: arrows, normal ER cisternae morphology; arrowheads, dilated ER cisternae; magnificatio*n =* ×1900; scale bar = 2 µm. (C) Pearson correlation analysis was performed on the arbitrary value of quantified data plotted against gestational age, with *p ≤* 0.05 considered significant; power regression lines were fitted to display the relationship; diamonds, PE samples; squares, NTC samples; ****p < 0.0001; ***p < 0.001

We recently demonstrated a high degree of ER stress in IUGR placentae, and that this was more pronounced in IUGR cases complicated with pre-eclampsia 26. In the current study, we further investigated the degree of ER stress in early-onset PE (<34 weeks; early-PE), late-onset PE (≥34 weeks; late-PE) and NTC placentae, using both western blotting analysis of UPR pathways and electron microscopy. Severity of ER stress can be adduced directly from alterations in UPR pathways, including changes in phosphorylation, increases in ER chaperone protein abundance, and protein and mRNA cleavage. Hence, phosphorylation of eIF2*α* and IRE1*α*, cleavage of ATF6 [ATF6 (p50)] and levels of GRP78, GRP94 and XBP-1 were investigated. Comparison of these markers revealed a significant increase of activation of the UPR pathways in the early-PE placentae, with elevated ATF6 (p50), P-IRE1*α*, XBP-1, GRP78 and GRP94 in comparison to both late-PE and NTC samples (Figure 1A; see also supplementary material, Figure S1A). The data for P-eIF2*α* were not significantly different amongst the groups, but this was due to an outsider in one of the late-PE samples (see supplementary material, Figure S1). By contrast, no difference in activation of the pathways was observed between late-PE placentae and the normotensive term controls. Ultrastructural analysis revealed dilatation of ER cisternae in early-PE (29 weeks), but not in late-PE (38 weeks) or NTC (39 weeks), placentae (Figure 1B), indicating loss of ER homeostasis only in the former.

### UPR activation is negatively correlated with gestational age at delivery in pre-eclamptic placentae

As the gestational age of the placentae studied ranged as a continuum from 24 to 39 weeks, we tested whether there was a correlation between the level of UPR activation and gestational age at delivery. A significant negative correlation was detected for several of the pathways; the coefficient of determination, *R*^2^, was highest for ATF6 (p50) at 0.73, and for other markers was in the range 0.54–0.34 (*p ≤* 0.0005) (Figure 1C). P-eIF2*α* was an exception but, after excluding the outsider, *R*^2^ for P-eIF2*α*/eIF2*α* was 0.22 (*p ≤* 0.0085; see supplementary material, Figure S1).

### P-AMPK*α*, P-p38 kinase and HSP70 are negatively correlated with gestational age at delivery in pre-eclamptic placentae

**Figure 2:**
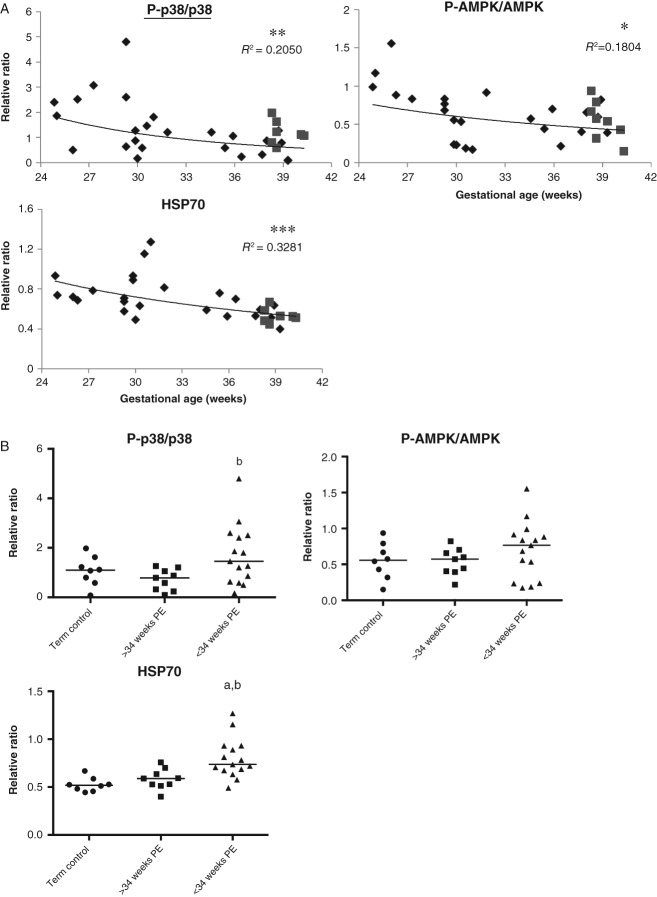
A negative correlation between activated p38 kinase, AMPK*α*, HSP70 and gestational age of PE placentae. (A) Pearson correlation analysis was performed on the arbitrary value of quantified data plotted against gestational age, with *p ≤* 0.05 considered significant; power regression lines were fitted to display the relationship: diamonds, PE samples; squares, NTC samples; ***p < 0.001; **p < 0.01; *p < 0.05. (B) Comparison among early-PEs, late-PEs and NTCs; the intensity of bands was quantified and normalized with *β*-actin: data are presented as medians; a non-parametric Kruskal–Wallis test with Dunn's multiple comparison test was used, with *p ≤* 0.05 considered significant; a, b, significant differences between < 34 weeks PE and NTC or ≥ 34 weeks PE, respectively

In the literature, there are conflicting reports regarding the activation of other placental stress signalling pathways in PE 30,31. However, these studies have only compared samples from either early- or late-onset PEs to normotensive pregnancies. Therefore, it was of interest to examine changes in these stress pathways in relationship to gestational age at delivery. We investigated the phosphorylation level of the energy-sensing kinase, AMPK*α*; MAPK stress pathway, p38 kinase and JNK; and the levels of the HSP family members HSP90, HSP70 and HSP27. Quantification showed a significant negative correlation between gestational age and the ratios of P-p38/p38 and P-AMPK*α*/AMPK*α* [*R*^2^ = 0.2050 (*p =* 0.0093) and *R*^2^ = 0.1804 (*p =* 0.0154), respectively]; and HSP70 [*R*^2^ = 0.3281 (*p =* 0.0006)] (Figure 2A). No correlation was observed for P-JNK/JNK, HSP90 and HSP27, with *R*^2^ values < 0.0388 (see supplementary material, Figure S2). However, when the three groups were compared using the Kruskal–Wallis test, only HSP70 was significantly higher in early-PE than both late-PE and NTC, while the difference in P-p38:p38 ratio was only significant between early-PE and late-PE (Figure 2B). No difference was observed among the three groups for P-AMPK*α*/AMPK*α*, P-JNK/JNK, HSP90 and HSP27 (Figure 2B; see also supplementary material, Figure S2).

**Figure 3:**
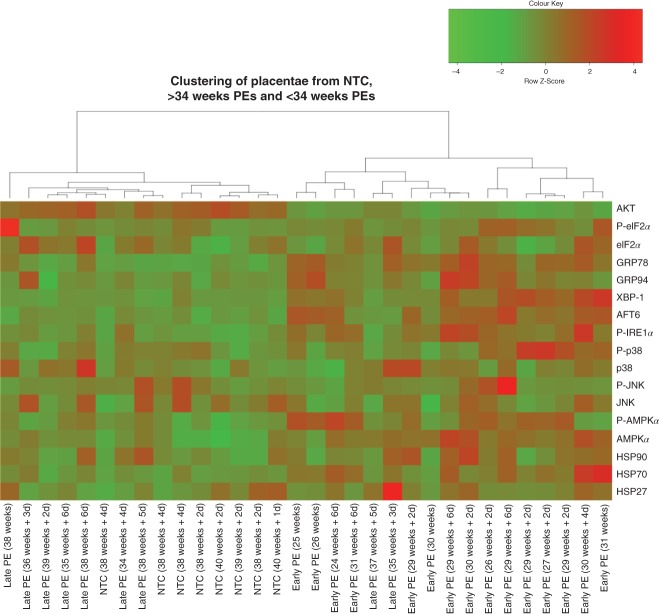
Placentae from early-PE cluster separately from the majority of late-PEs and NTCs, based on the levels of stress-response proteins. Cluster analysis of western blot data was performed using Ward's agglomerative method: d, days

To further investigate the differential stress signalling pathways in the early-PE and late-PET placentae, a cluster analysis using all the data was performed. The data can be clustered into two major branches, one containing all the early-PE samples and two late-PE (35 and 37 weeks) cases, while the other contains all remaining late-PE and all NTC samples (Figure 3). These results clearly demonstrate that placentae from early-PE suffer from a high degree of physiological/cellular stress, whereas, in contrast, these stress pathways are not activated in the late-PE placentae. The latter are indistinguishable from the NTC.

### ER stress severity does not alter across gestational age during normal placental development

**Figure 4:**
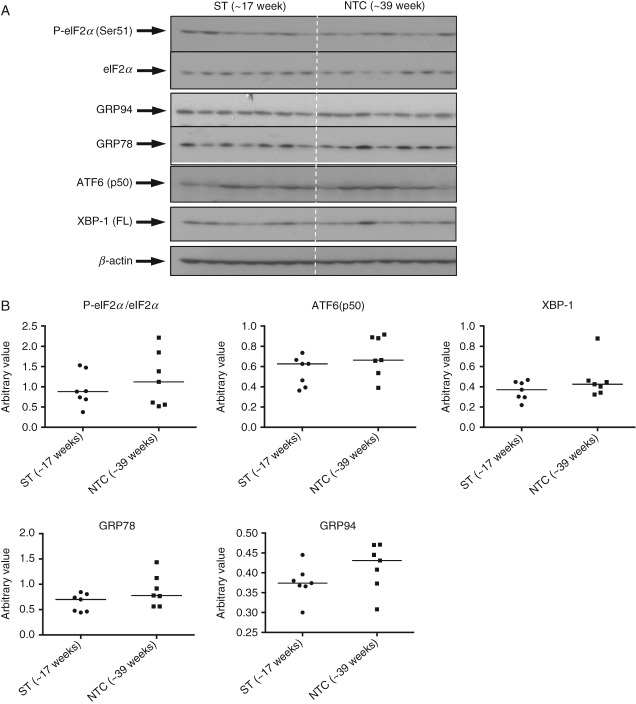
No change of UPR activation in second-trimester placentae compared to normotensive term placentae. (A) Tissue lysates from seven second-trimester (ST) and seven NTC placentae were resolved in SDS–PAGE before probing with antibodies against ER stress markers. (B) The intensity of bands was quantified and normalized with *β*-actin; phosphorylation status is presented as the ratio between phosphorylated and total protein: data are medians for seven placentae/group; no significant changes were observed in any UPR pathway

To test for effects of gestational age on ER stress levels, we compared 14 placentae from late second-trimester (∼17 weeks) and NTC (∼39 weeks). We also examined PTC (∼29 weeks, *n =* 7). It should be noted that the PTCs were from vaginal deliveries following spontaneous labour, with acute chorioaminionitis or other pathological conditions, as pre-term healthy placentae delivered by elective caesarean section are almost impossible to obtain. No difference in the activation of UPR pathways was observed between second-trimester and NTC placentae (Figure 4). By contrast, P-eIF2*α*, eIF2*α*, XBP-1 and GRP78 were increased significantly in PTC placentae, while GRP94 was decreased (see supplementary material, Figure S3 A, B). These results indicate that levels of ER stress are unlikely to alter substantially during normal placental development, whereas laboured pathological PTC placentae suffer from ER stress. Therefore, in this context, they cannot be considered appropriate controls.

### Severe, but not mild hypoxia–reoxygenation induces ER stress and reduces cell proliferation in BeWo cells

**Figure 5:**
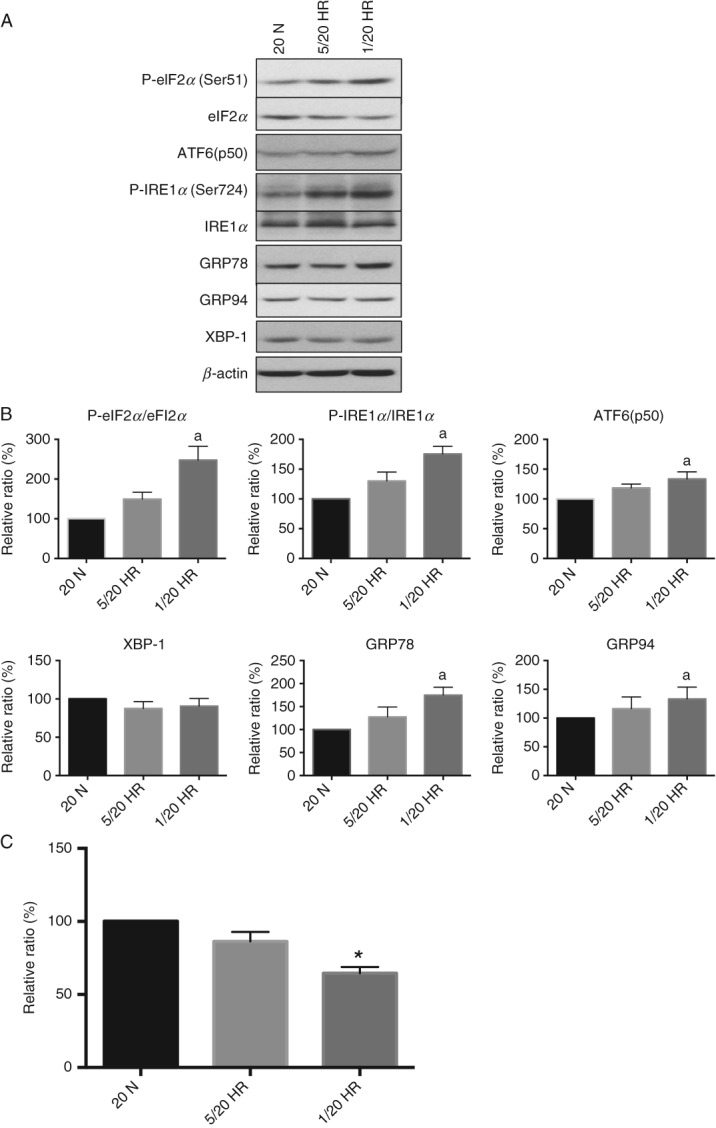
Severe but not mild hypoxia–reoxygenation activates the UPR and reduces proliferation of BeWo cells. (A) Cells were incubated under either 6 h repetitive cycles of 1% and 20% O_2_ (severe H/R) or 5% and 20% O_2_ (mild H/R) for 24 h. Cell lysates were collected and resolved in SDS–PAGE before probing with primary antibodies specific for UPR markers; *β*-actin was used as the loading control. (B) Densitometry of bands expressed relative to normal controls; phosphorylation status is presented as the ratio between phosphorylated and total protein: data are mean ± SEM for four independent experiments; a, significance at *p <* 0.05 compared to normoxic controls (20 n). (C) Cells used for cell proliferation assay were subjected to 48 h H/R challenge and the numbers compared to the 20% O_2_ normoxic controls (100%): data are mean ± SEM from four independent experiments; *p < 0.05

Placental malperfusion resulting in oxidative damage is considered one of the major contributors to the pathophysiology of early-onset PE 21,22. Our previous study demonstrated that hypoxia–reoxygenation can strongly induce ER stress in trophoblastic cells 26. Therefore, an *in vitro* cell culture model was used to investigate how different severities of hypoxia–reoxygenation (5/20 and 1/20 H/R) activate the UPR pathways in trophoblast-like cells, and their impact on cell proliferation. Primary trophoblast cells display a high degree of ER stress following isolation and culture (see supplementary material, Figure S4) and are post-mitotic. Therefore, the villous-like tophoblastic cell line BeWo was used in this study. As the majority of ER stress *in vivo* occurs in the syncytiotrophoblast, syncytialization of the BeWo cells was induced prior to exposure to H/R. We observed an H/R severity-dependent increase of activation of the UPR pathways (Figure 5A); the majority were significantly activated following 1/20 H/R but not after 5/20 H/R (Figure 5B). Other stress markers, including P-AMPK*α*/AMPK*α* and P-p38/p38 were also significantly increased following 1/20 H/R (eight- and 16-fold respectively, data not shown).

ER stress has been demonstrated to slow cell proliferation in trophoblast-like cells 29. Therefore, we investigated the proliferation rate of BeWo cells under H/R conditions. There was no significant reduction following 5/20 H/R, but the proliferation rate was reduced by 35% following 1/20 H/R (Figure 5C).

### Pro-inflammatory cytokines have only a subtle effect in the activation of ER stress in BeWo cells

**Figure 6:**
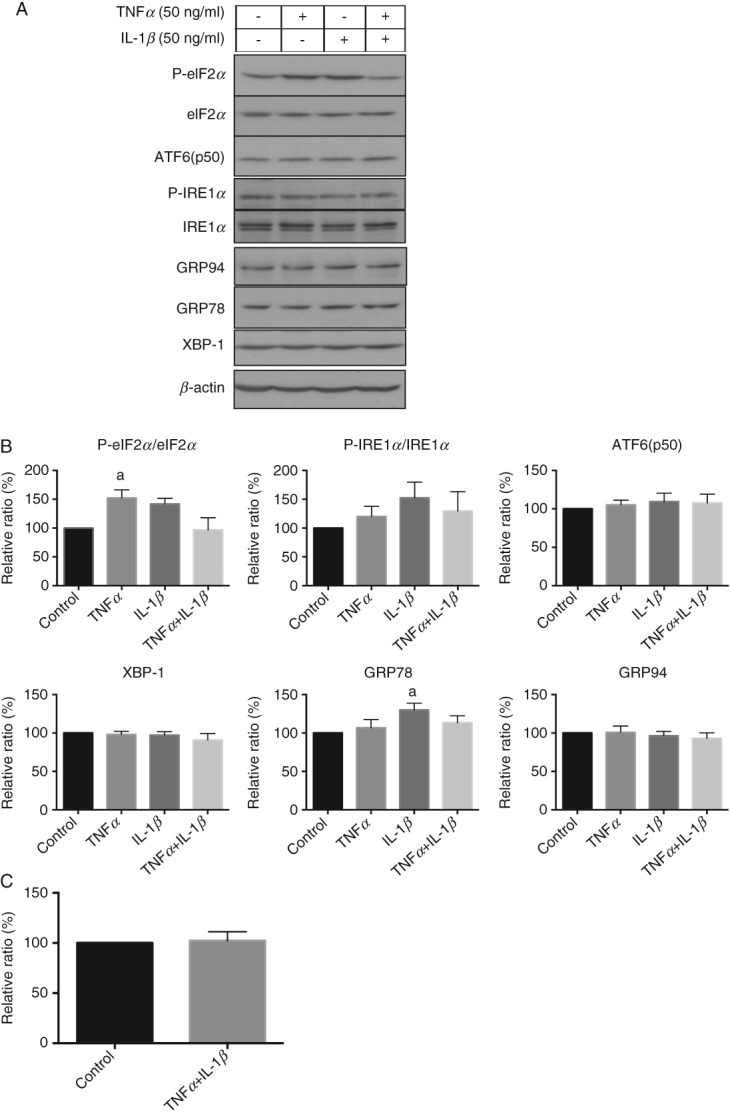
Pro-inflammatory cytokines, TNF*α* and IL-1*β*, have a subtle effect on UPR pathways but do not affect cell proliferation. (A) Cells were treated with TNF*α* (50 ng/ml), IL-1*β* (50 ng/ml) or both for 24 h. Cell lysates were subjected to western blotting analysis with antibodies specific for UPR markers; *β*-actin was used as the loading control. (B) Densitometry of bands expressed relative to normal controls; phosphorylation status is presented as the ratio between phosphorylated and total protein: data are mean ± SEM for five independent experiments; a, significance at *p <* 0.05 compared to non-treatment controls (Control). (C) Cells used for the cell proliferation assay were subjected to 48 h incubation and the numbers compared to the non-treatment controls (100%): data are mean ± SEM from four independent experiments

The concentration of the circulating pro-inflammatory cytokine TNF*α* is increased two-fold in pre-eclampsia, while IL-1*β* is elevated during normal pregnancy but does not increase further in PE 32. Both of these cytokines induce ER stress in other cell systems 33,34. Therefore, we investigated whether they were able to induce ER stress in the syncytialized BeWo cells. The effects of TNF*α* and IL-1*β* on activation of the UPR pathways was very subtle in comparison to the H/R challenge, with approximately 1.5- and 1.2-fold increases in P-eIF2*α/*eIF2 and GRP78, respectively (Figure 6A, B). TNF*α*, but not IL-1*β*, also activated phosphorylation of p38 kinase (1.7-fold; see supplementary material, Figure S5). Furthermore, TNF*α* and IL-1*β* treatment did not affect BeWo cell proliferation (Figure 6C).

### Down-regulation of AKT protein in both < 34 weeks PE placentae and severely stressed BeWo cells

**Figure 7:**
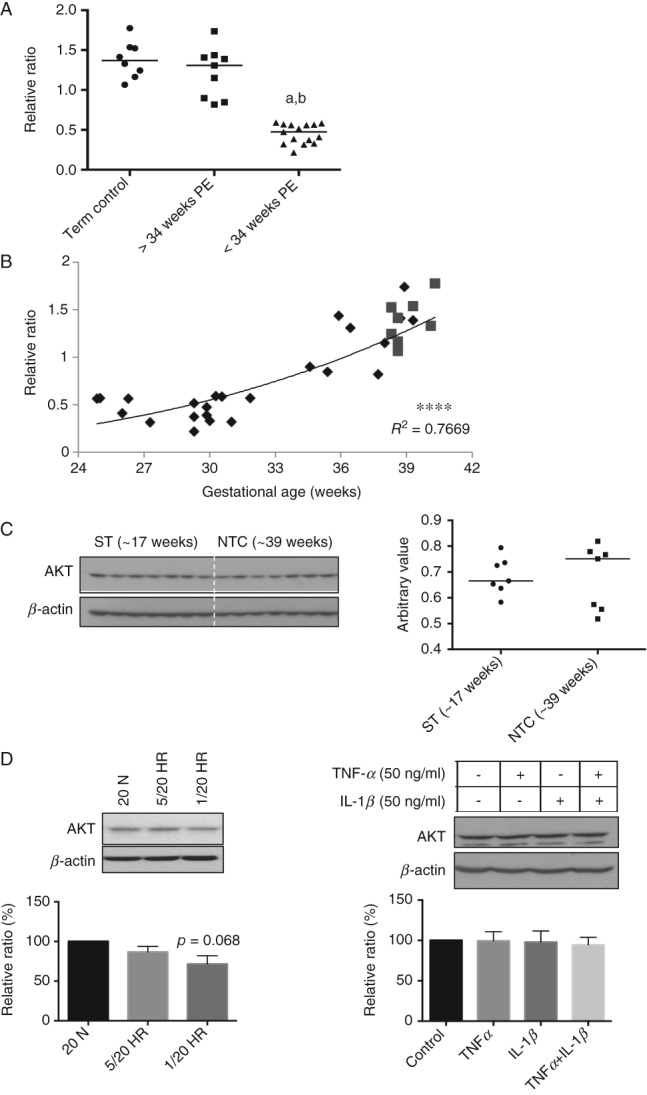
Down-regulation of AKT in the < 34 weeks PE placentae and severe H/R-treated cells, but not in the ≥ 34 weeks PE placentae, second-trimester, mild H/R-treated and TNF*α* + IL-1*β*-treated cells. (A) Comparison among early-PEs, late-PEs and NTCs: data are presented as medians; a non-parametric Kruskal–Wallis test with Dunn's multiple comparison test was used, with *p ≤* 0.05 considered significant; a, b, significant differences between < 34 weeks PE and term control or ≥ 34 weeks PE, respectively. (B) Correlation analysis on the arbitrary value of quantified data was plotted against gestational age; a Pearson correlation was performed, with *p ≤* 0.05 being considered significant; power regression lines were fitted to display the relationship: diamonds, PE samples; squares, NTC samples; ****p < 0.0001. (C) AKT protein level was analysed in the second-trimester placentae and normotensive term controls by western blot. (D) H/R or TNF*α* and IL-1*β* treatment was performed and western blotting was used to analyse AKT protein level; *β*-actin was used as the loading control: data are mean ± SEM for four or five independent experiments; *p =* 0.068 compared to normoxic control

AKT signalling is a central regulatory pathway for cell proliferation, growth and survival. We have previously demonstrated that ER stress inhibits translation of AKT in IUGR placentae 26. In the present study, AKT was reduced in the < 34 weeks PE placentae but not in the ≥ 34 weeks samples when compared to NTC (Figure 7A). There was a strong positive correlation (*R*^2^ = 0.7669) between AKT protein level and gestational age of delivery in PE placentae (Figure 7B). No difference was observed between the second-trimester and NTC placentae (Figure 7C). Furthermore, we observed a trend in reduction of AKT (*p =* 0.068) in BeWo cells following 1/20 H/R, but not after 5/20 H/R or treatment with TNF*α* and/or IL-1*β* (Figure 7D).

## Discussion

To our knowledge, this is the first study to investigate the placental stress signalling pathways in patients with pre-eclampsia across the gestational age range at the time of delivery determined by the clinical needs of the mother and fetus. Activation of UPR pathways, p38 kinase and AMPK*α* phosphorylation showed strong negative correlations with gestational age at delivery (Figures 1, 2); the earlier the gestational age, the higher the level of stress. Indeed, those placentae delivered after 34 weeks were indistinguishable from the normotensive term controls. The difference in placental stress is unlikely to be attributable to gestational age, as we were unable to detect any difference between the second-trimester and term-control placentae. Obtaining suitable healthy age-matched pre-term controls is almost impossible; those pre-term placentae available to us showed increased activation in some stress-response pathways, but this is likely to have been induced by the pathology stimulating their delivery or the labour process. Notably, we have previously demonstrated that labour is a strong inducer of placental oxidative stress, which in turn activates ER stress *in vitro* (Figure 5) 25,35.

The question arises as to whether the high levels of placental stress may contribute to the pathophysiology of early-onset pre-eclampsia, rather than being a consequence of the inflammatory maternal milieu. Our *in vitro* results suggest the former, since pro-inflammatory cytokines had little impact on activation of UPR pathways in trophoblast-like cells. Rather, we were able to mimic our *in vivo* findings by exposing the cells to severe fluctuations in oxygenation, which have been postulated to occur following abnormal placentation. How the placental stress may contribute to the syndrome of early-onset pre-eclampsia is uncertain, but high levels of activation of the UPR can lead to inflammatory responses by diverse pathways 36. To summarize, our findings are consistent with the concept that poor placentation in early pregnancy leads to malperfusion and placental stress, which in turn causes the release of pro-inflammatory cytokines and/or anti-angiogenic factors that induce activation of the maternal endothelial cells. Furthermore, we confirm that late-onset pre-eclampsia is usually associated with normal placental function 4,6.

It is well recognized that fetal growth restriction is more commonly present with early-onset or ‘placental’ PE than with late-onset or ‘maternal’ PE. AKT signalling is crucial for placental and fetal growth. IUGR placentae exhibit low AKT 26, and *Akt1*-deficient mice display an IUGR phenotype 26,37. AKT was notably lower in the PE placentae delivered < 34 weeks than ≥ 34 weeks, consistent with the higher risk of IUGR in early-onset PE (Figure 7A). Again, the difference could not be due to gestational age, as AKT protein level was relatively constant between 17 weeks and term (Figure 7C). In an *in vitro* study, ER stress-induced inhibition of protein synthesis caused a reduction in AKT, and was associated with reduced trophoblast-like cell proliferation 25,26. In this study, severe, but not mild, hypoxia–reoxygenation reduced BeWo cell proliferation and was associated with a reduction in AKT (Figures 5C, 7D). By contrast, the pro-inflammatory cytokines TNF*α* and IL-1*β* had no effect on AKT level or cell proliferation (Figures 6C, 7D). As reduced placental growth precedes fetal growth restriction in human pregnancy 38,39, this mechanism may contribute to the IUGR associated with early-onset PE.

**Figure 8:**
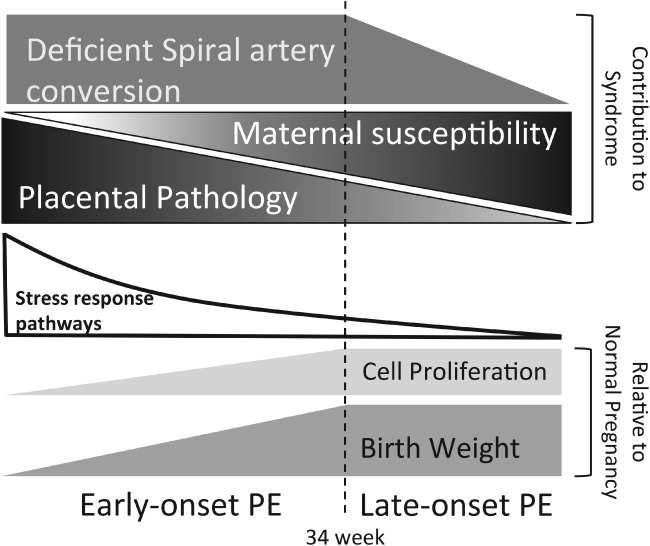
Schematic diagram illustrating how our findings fit into a model integrating the pathophysiology (degree of spiral artery conversion), different potential aetiologies (placental pathology and maternal susceptibility) and clinical outcomes of pre-eclampsia at different gestational ages

In conclusion, our data provide the first molecular evidence of a difference in activation of placental UPR signalling pathways and a concomitant reduction in AKT between patients with pre-eclampsia delivered due to clinical necessity < 34 weeks and those delivered ≥ 34 weeks. These findings support the concept that cases of early-onset pre-eclampsia are predominantly due to placental pathology, while those of late-onset are likely the result of increased maternal sensitivity to the pro-inflammatory environment of pregnancy, due to metabolic or other disturbances. These two aetiologies are not mutually incompatible, and there is likely to be overlap around 32–35 weeks and possibly beyond (Figure 8). Separating pre-eclampsia into sub-types will assist in understanding the underlying pathophysiology and in developing appropriate therapeutic interventions.

## Abbreviations

AKT, protein kinase B; AMPK*α*, AMP-activated protein kinase-*α*; ATF6, activating transcription factor 6; eIF2*α*, eukaryotic initiation factor 2 subunit-*α*; GRP78, glucose regulated protein 78; GRP94, glucose-regulated protein 94; HR, hypoxia–reoxygenation; IL-1*β*, interleukin 1*β*; MAPK, mitogen-activated protein kinase; IRE1*α*, inositol requiring protein kinase; IUGR, intrauterine growth restriction; PE, pre-eclampsia; TNF*α*, tumour necrosis factor-*α*; UPR, unfolded protein response; XBP-1, X-box transcription factor.

## Supplementary materials On The Internet

The following supplementary material may be found in the online version of this article:

Supplementary information on materials

Figure S1. (A) Western blot images of all ER stress markers, p38 kinase, JNK and HSPs studied in the Early-PE, Late-PE and NTC

Figure S2. JNK MAPK kinase and heat shock protein family members and gestational age at delivery of PE placentae

Figure S3. Subtle increase of ER stress in the preterm 'control' placentae compared to second-trimester and normotensive term placentae

Figure S4. Higher ER stress in cultured primary trophoblast cells than in BeWo cells

Figure S5. TNF*α*, but not IL-1*β*, activates phosphorylation of p38 kinase

Table S1. Detailed clinical characteristics of each individual placenta used in this study, except second-trimester placentae

## Supporting Information

Appendix S1 Supplementary information on materials.

Figure S1 (A) Western blot images of all ER stress markers, p38 kinase, JNK and HSPs studied in the early-PEs, late-PEs and NTCs. Due to the limited number of samples that could be loaded on a single gel, western blotting analysis on 32 samples was performed on two groups of samples: the first contained 19 PE placentae, of which 15 were early-PEs and four were late-PEs; the second contained 16 samples, of which eight were late-PEs and eight were NTCs; three late-PE samples were duplicated in both groups in order to allow inter-gel comparison. (B) Removal of an outsider in the late-PE samples revealed a significant negative correlation between the ratio of PeIF2*α*:eIF2*α* with gestational age of delivery, and is also higher in the early-PE compared to the late-PE placentae. We have previously shown that phosphorylation of eIF2*α* is very sensitive to the delivery–collection interval, which could be an explanation for the outsider

Figure S2 JNK MAPK kinase and heat shock protein family members and gestational age at delivery of PE placentae. No significant correlation existed, and there was no significant difference between the groups. Pearson's correlation was used to test for significance and a power regression line was added to show the relationship; a paired non-parametric Kruskal–Wallis test with Dunn's multiple comparison test was used to show differences among the three groups. (A) JNK; (B) HSP90 and HSP27. In the correlation graphs, diamonds indicate PE samples, while squares indicate NTC samples

Figure S3 A subtle increase of ER stress in the preterm 'control' placentae compared to second-trimester and normotensive term placentae. (A) Tissue lysates from seven second-trimester (ST), seven NTC and seven PTC placentae were resolved by SDS–PAGE before probing with antibodies against the same ER stress markers as in FigureB. Densitometry of bands was expressed relative to normal controls (100%); phosphorylation status is presented as the ratio between phosphorylated and total protein, both normalized to *β*-actin: data are median for seven placentae/group; a, b, significance *p ≤* 0.05 compared to ST and NTC, respectively

Figure S4 Higher ER stress in cultured primary trophoblast cells than in BeWo cells. Primary trophoblast cells were isolated from the first-trimester placentae as described by Trundley. Both BeWo cells and freshly isolated primary trophoblast cells were cultured under either 10% (control) or 1% (hypoxia) O_2_ for 24 h prior for western blotting analysis with ER stress markers; *β*-actin was used as loading control

Figure S5 TNF*α*, but not IL-1*β*, activates phosphorylation of p38 kinase. BeWo cells were treated with TNF*α* (50 ng/ml) and/or IL-1*β* (50 ng/ml) for 24 h. (A) Images of a representative blot of P-p38, p38 and *β*-actin. (B) Densitometry of bands expressed relative to normal controls as 100%; phosphorylation status is presented as the ratio between phosphorylated and total protein: data are mean ± SEM for five independent experiments; a, significance at *p ≤* 0.05 compared to control

Table S1 Detail clinical characteristics of pre-eclamptic, term- and preterm- control placentas
