# Supplementary material for: Differential activation of placental unfolded protein response pathways implies heterogeneity in causation of early- and late-onset pre-eclampsia
Source: J Pathol. 2014 Aug 6;234(2):262–76. doi: 10.1002/path.4394 (PMC4277692; doi:10.1002/path.4394)
Supplement: Appendix S1 — Supplementary information on materials. [file path0234-0262-sd1.doc]

**<Supplementary material>**

+A: **Supplementary information on materials**

All chemicals and tissue culture reagents were purchased from Sigma-Aldrich and Invitrogen, respectively, except where otherwise mentioned. The antibodies anti-phospho-eIF2*α* (Ser51), anti-eIF2*α*, anti-phospho-p38 MAPK (Thr180/Tyr182), anti-p38 kinase, anti-phospho-SAPK/JNK (Thr183/Tyr185), anti-SAPK/JNK, P-AMPK*α*(Thr172), AMPK*α* and anti-HSP27 were from Cell Signaling Technology (NEB, Hitchin, UK). Anti-IRE1*α* (phospho S724), anti-IRE1α, anti-ATF6, XBP-1, GRP94 and anti-KDEL were from Abcam (Cambridge, UK). Anti-HSP90 and anti-HSP70 were from Enzo Life Science (Exeter, UK). Anti-GRP78 was from Transduction Laboratories (BD Biosciences, Oxford, UK) and anti-*β*-actin was from Sigma-Aldrich.
