# Supplementary figures and images for: Differential activation of placental unfolded protein response pathways implies heterogeneity in causation of early- and late-onset pre-eclampsia
Source: J Pathol. 2014 Aug 6;234(2):262–76. doi: 10.1002/path.4394 (PMC4277692; doi:10.1002/path.4394)

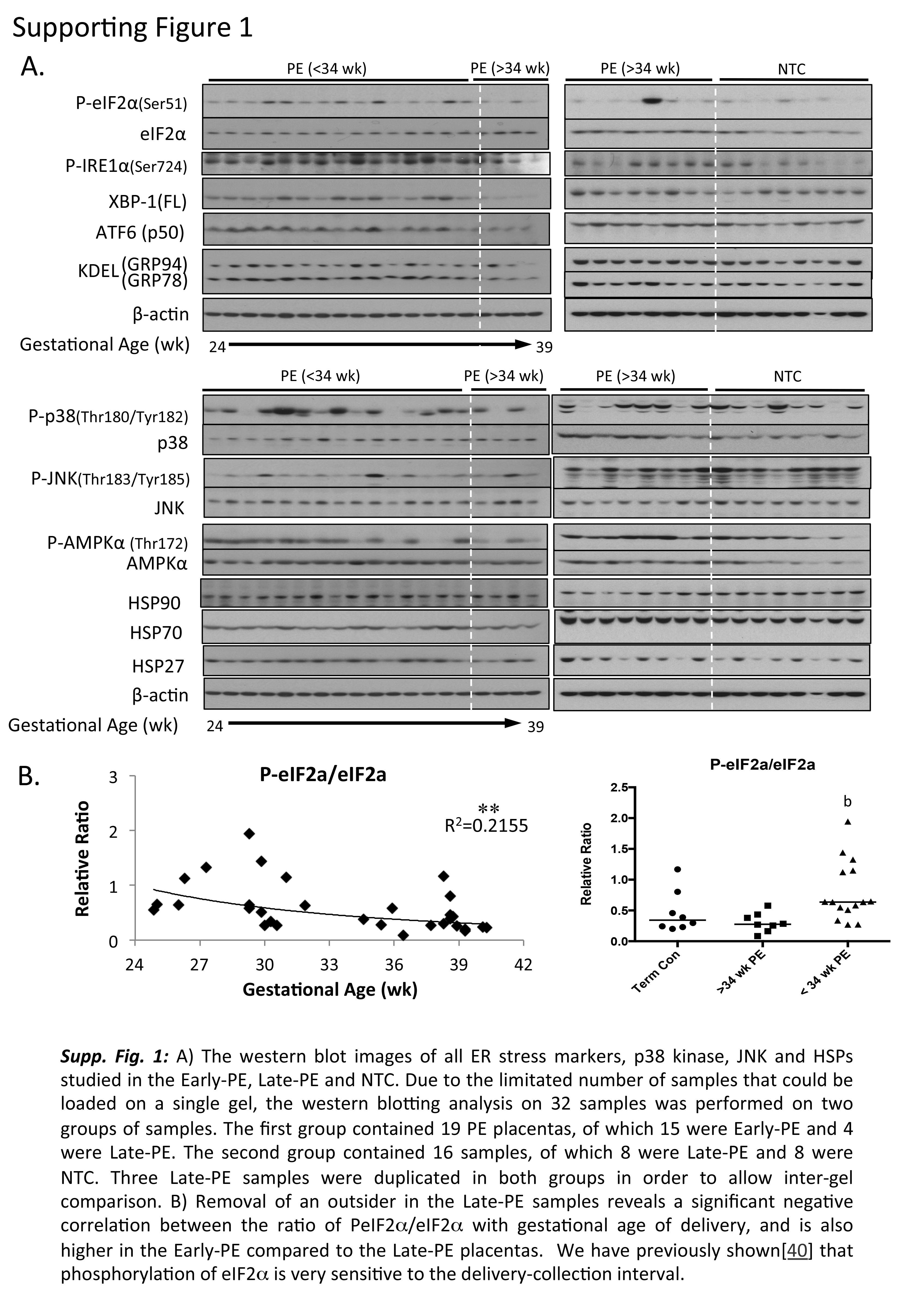

Supplement: Figure S1 — (A) Western blot images of all ER stress markers, p38 kinase, JNK and HSPs studied in the early-PEs, late-PEs and NTCs. Due to the limited number of samples that could be loaded on a single gel, western blotting analysis on 32 samples was performed on two groups of samples: the first contained 19 PE placentae, of which 15 were early-PEs and four were late-PEs; the second contained 16 samples, of which eight were late-PEs and eight were NTCs; three late-PE samples were duplicated in both groups in order to allow inter-gel comparison. (B) Removal of an outsider in the late-PE samples revealed a significant negative correlation between the ratio of PeIF2α:eIF2α with gestational age of delivery, and is also higher in the early-PE compared to the late-PE placentae. We have previously shown that phosphorylation of eIF2α is very sensitive to the delivery–collection interval, which could be an explanation for the outsider [file path0234-0262-sd2.tif]

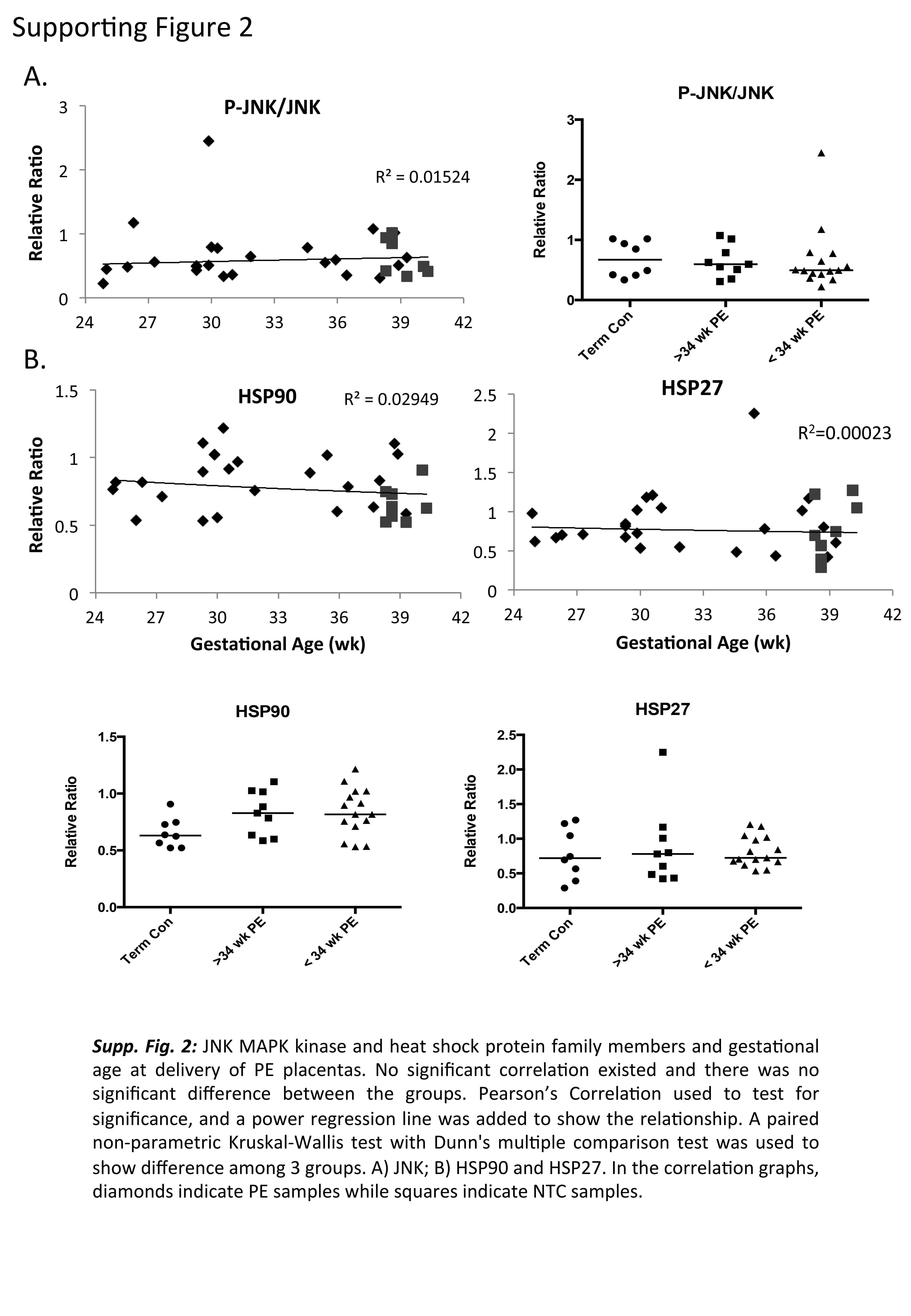

Supplement: Figure S2 — JNK MAPK kinase and heat shock protein family members and gestational age at delivery of PE placentae. No significant correlation existed, and there was no significant difference between the groups. Pearson's correlation was used to test for significance and a power regression line was added to show the relationship; a paired non-parametric Kruskal–Wallis test with Dunn's multiple comparison test was used to show differences among the three groups. (A) JNK; (B) HSP90 and HSP27. In the correlation graphs, diamonds indicate PE samples, while squares indicate NTC samples [file path0234-0262-sd3.tif]

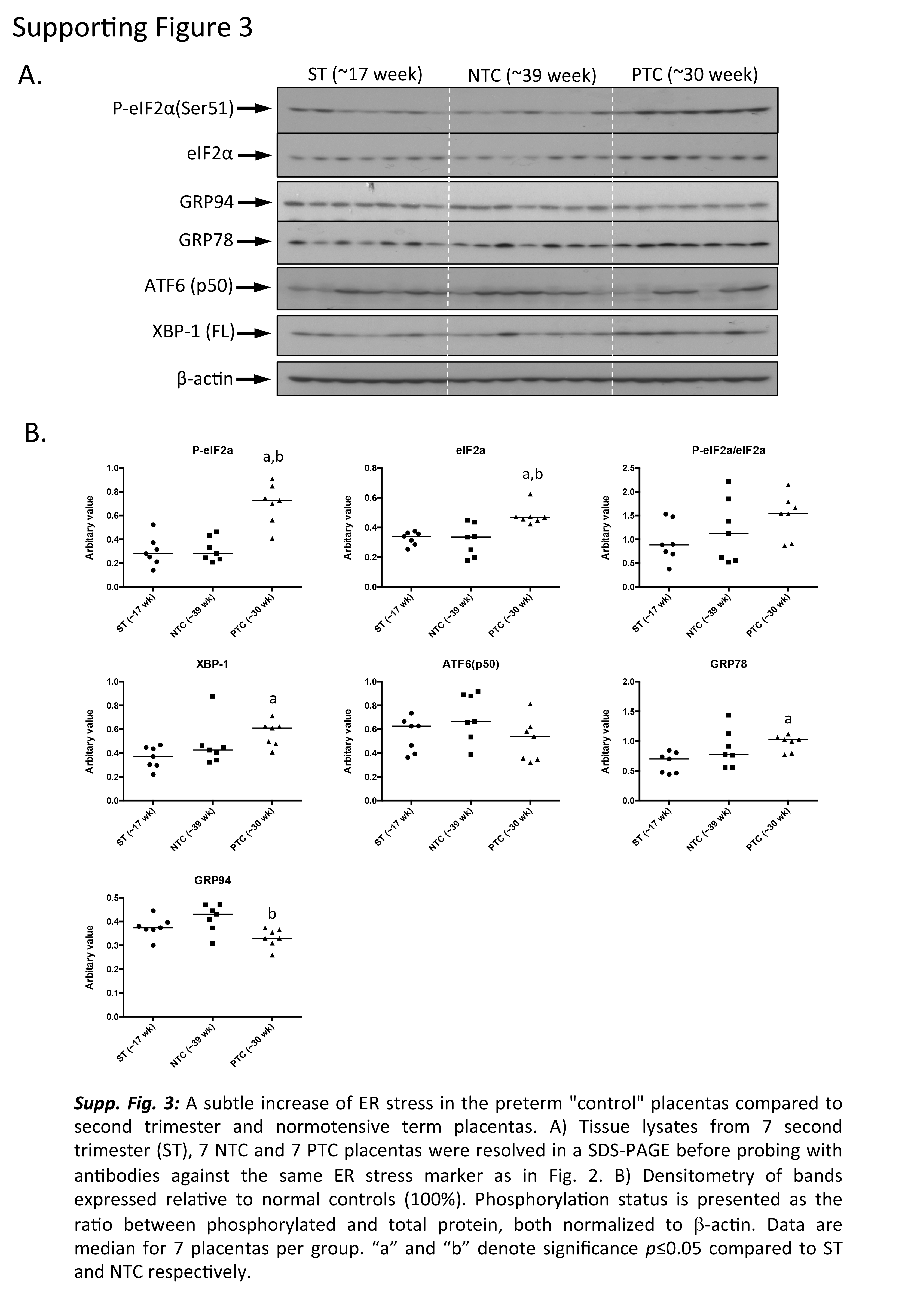

Supplement: Figure S3 — A subtle increase of ER stress in the preterm 'control' placentae compared to second-trimester and normotensive term placentae. (A) Tissue lysates from seven second-trimester (ST), seven NTC and seven PTC placentae were resolved by SDS–PAGE before probing with antibodies against the same ER stress markers as in FigureB. Densitometry of bands was expressed relative to normal controls (100%); phosphorylation status is presented as the ratio between phosphorylated and total protein, both normalized to β-actin: data are median for seven placentae/group; a, b, significance p ≤ 0.05 compared to ST and NTC, respectively [file path0234-0262-sd4.tif]

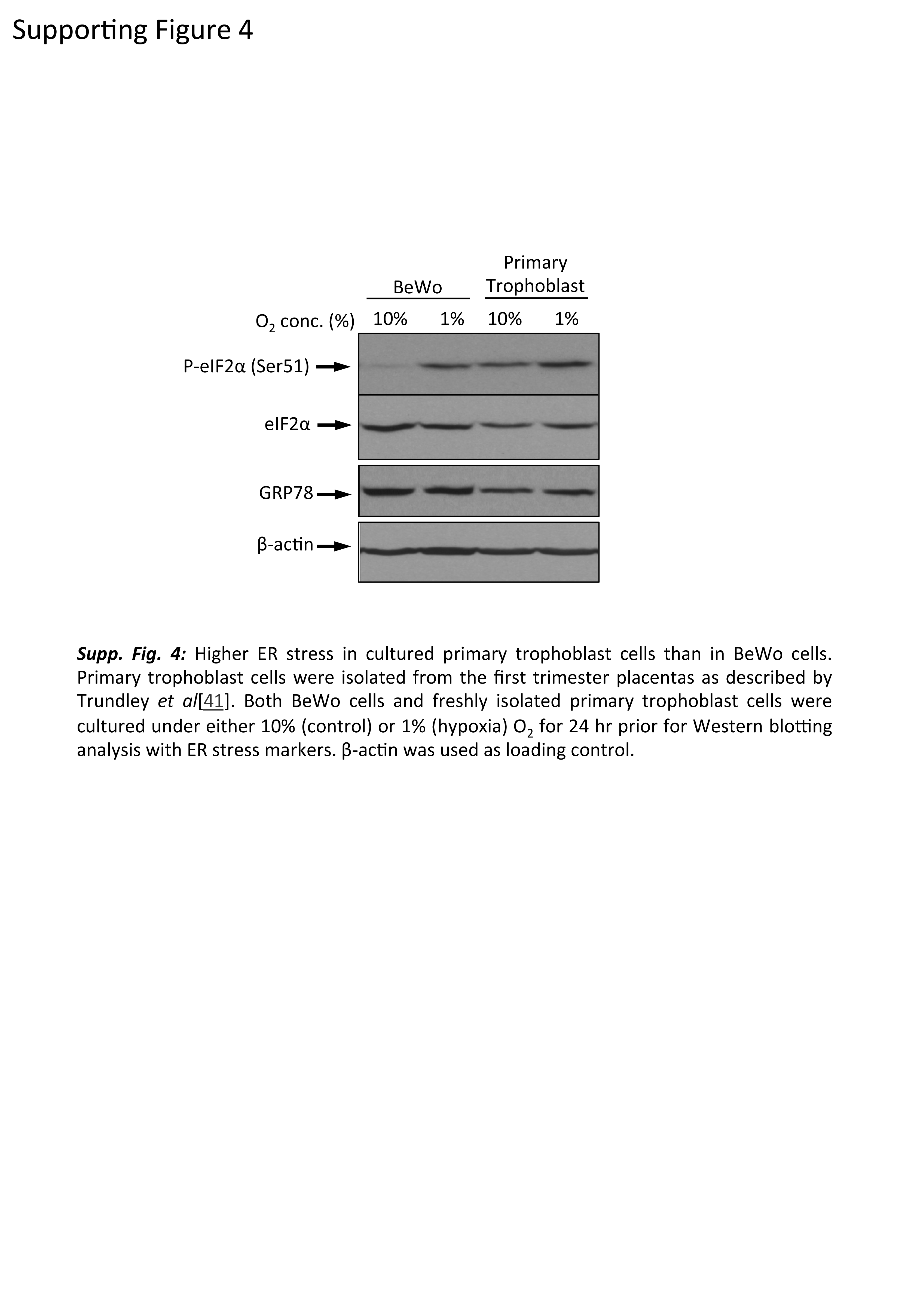

Supplement: Figure S4 — Higher ER stress in cultured primary trophoblast cells than in BeWo cells. Primary trophoblast cells were isolated from the first-trimester placentae as described by Trundley. Both BeWo cells and freshly isolated primary trophoblast cells were cultured under either 10% (control) or 1% (hypoxia) O2 for 24 h prior for western blotting analysis with ER stress markers; β-actin was used as loading control [file path0234-0262-sd5.tif]

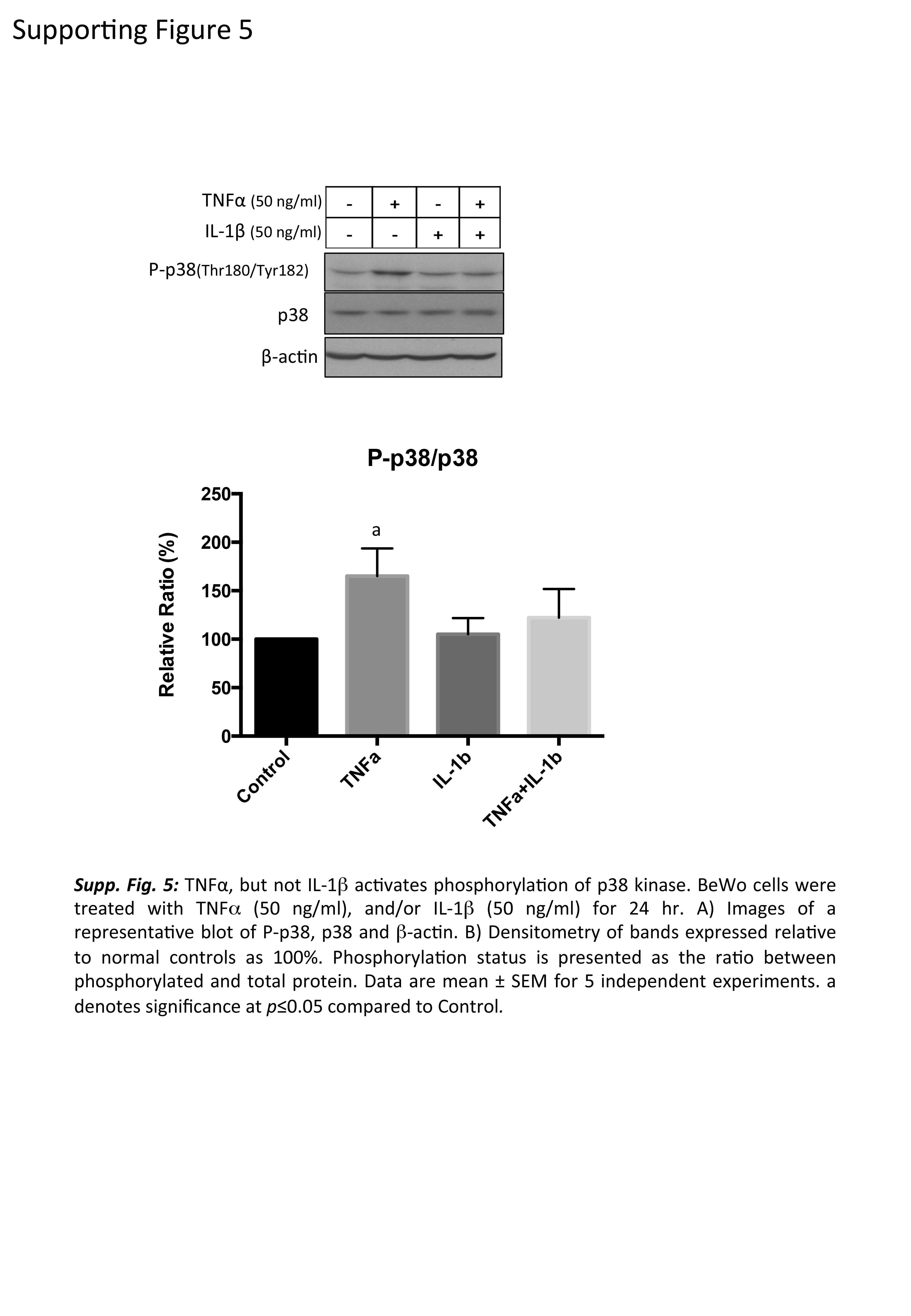

Supplement: Figure S5 — TNFα, but not IL-1β, activates phosphorylation of p38 kinase. BeWo cells were treated with TNFα (50 ng/ml) and/or IL-1β (50 ng/ml) for 24 h. (A) Images of a representative blot of P-p38, p38 and β-actin. (B) Densitometry of bands expressed relative to normal controls as 100%; phosphorylation status is presented as the ratio between phosphorylated and total protein: data are mean ± SEM for five independent experiments; a, significance at p ≤ 0.05 compared to control [file path0234-0262-sd6.tiff]
