# Supplementary material for: Differential activation of placental unfolded protein response pathways implies heterogeneity in causation of early- and late-onset pre-eclampsia
Source: J Pathol. 2014 Aug 6;234(2):262–76. doi: 10.1002/path.4394 (PMC4277692; doi:10.1002/path.4394)
Supplement: Table S1 — Detail clinical characteristics of pre-eclamptic, term- and preterm- control placentas [file path0234-0262-sd7.doc]

**Supporting table 1:** Detail clinical characteristics of pre-eclamptic, term- and preterm- control placentas

| Gest age (wk) | Maternal Age | BMI | Proteinuria (mg/L) | BP syst | BP diast | Fet weight (g) | Birth Percentile | Pl weight (g) |
| --- | --- | --- | --- | --- | --- | --- | --- | --- |
| ***Preeclamptic Placentas*** | | | | | | | | |
| 24+6 | 34 | 24.1 | >1000 | 153 | 97 | 428 | <10% | 135 |
| 25 | 37 | 22.5 | >5000 | 180 | 105 | 533 | <10% | 120 |
| 26 | 31 | 34.4 | 6900 | 177 | 101 | 792 | 10-50% | 330 |
| 26+2 | 29 | 22.1 | 8160 | 168 | 108 | 792 | 10-50% | 182 |
| 27+2 | 31 | 31.6 | 115 | 140 | 96 | 788 | 10-50% | - |
| 29+2 | 26 | 28.4 | >5000 | 150 | 95 | 705 | <5% | 180 |
| 29+2 | 39 | 26.8 | >5000 | 197 | 103 | 980 | 10-50% | 300 |
| 29+2 | 35 | 31.6 | 242 | 186 | 104 | 1000 | 10-50% | 280 |
| 29+6 | 29 | 23.2 | 11600 | 155 | 101 | 1245 | 10-50% | 325 |
| 29+6 | 29 | 21.1 | 5200 | 179 | 114 | 1320 | 10-50% | - |
| 30 | 30 | 21.2 | 5800 | 160 | 100 | 836 | <10% | 245 |
| 30+2 | 27 | 32.6 | 117 | 165 | 93 | 1115 | 10-50% | 310 |
| 30+4 | 29 | 37.1 | 7121 | 148 | 97 | 1210 | 10-50% | 310 |
| 31 | 37 | 25.2 | 9600 | 160 | 90 | 1485 | 10-50% | 270 |
| 31+6 | 24 | 27.6 | 1350 | 151 | 90 | 1648 | 10-50% | 390 |
| ***34+4*** | ***29*** | ***23.6*** | ***7236*** | ***149*** | ***97*** | ***2195*** | ***10-50%*** | ***690*** |
| ***35+3*** | ***31*** | ***21.6*** | ***2928*** | ***153*** | ***102*** | ***1830*** | ***<10%*** | ***370*** |
| ***35+6*** | ***32*** | ***26.8*** | ***732*** | ***165*** | ***106*** | ***2630*** | ***50-90%*** | ***470*** |
| ***36+3*** | ***28*** | ***24.2*** | ***2500*** | ***159*** | ***107*** | ***2440*** | ***10-50%*** | ***600*** |
| ***37+5*** | ***37*** | ***23.1*** | ***>300*** | ***145*** | ***100*** | ***2650*** | ***10-50%*** | ***-*** |
| ***38*** | ***31*** | ***20.6*** | ***>300*** | ***128*** | ***75*** | ***3180*** | ***10-50%*** | ***-*** |
| ***38+5*** | ***26*** | ***24.6*** | ***>1000*** | ***140*** | ***105*** | ***4320*** | ***>95%*** | ***850*** |
| ***38+6*** | ***36*** | ***25.6*** | ***>300*** | ***140*** | ***80*** | ***2740*** | ***<10%*** | ***-*** |
| ***39+2*** | ***43*** | ***45*** | ***>5000*** | ***135*** | ***91*** | ***3470*** | ***50-90%*** | ***650*** |
| ***Term Control Placentas*** | | | | | | | | |
| *40+2* | *31* | *18.2* | *<300* | *111* | *68* | *4110* | *90-95%* | *-* |
| *39+2* | *32* | *25.9* | *<300* | *125* | *75* | *3370* | *10-50%* | *610* |
| *38+4* | *40* | *20.3* | *<300* | *104* | *63* | *3240* | *50-90%* | *640* |
| *38+2* | *32* | *30.1* | *<300* | *124* | *95* | *5220* | *>95%* | *880* |
| *38+2* | *25* | *29.7* | *<300* | *118* | *78* | *4200* | *>95%* | *740* |
| *38+4* | *29* | *23* | *<300* | *114* | *59* | *4330* | *>95%* | *800* |
| *40+1* | *29* | *20.4* | *<300* | *130* | *81* | *3760* | *50-90%* | *520* |
| *38+4* | *44* | *28.7* | *<300* | *120* | *70* | *3450* | *50-90%* | *560* |
| *Preterm Control Placentas* | | | | | | | | |
| **31* | *35* | *18.3* | *-* | *110* | *60* | *1630* | *50-90%* | *275* |
| **27* | *38* | *-* | *-* | *130* | *90* | *900* | *10-50%* | *183* |
| **33* | *27* | *21.6* | *-* | *110* | *70* | *2460* | *50-90%* | *356* |
| **27* | *20* | *20.8* | *-* | *126* | *84* | *1030* | *10-50%* | *215* |
| **32* | *27* | *20* | *-* | *110* | *62* | *2190* | *50-90%* | *331* |
| **26* | *31* | *24* | *-* | *102* | *65* | *830* | *10-50%* | *205* |
| **27* | *26* | *18.3* | *-* | *102* | *75* | *970* | *10-50%* | *232* |

Remarks: * indicates that placental tissues were collected from vaginal delivery and were complicated with a number of conditions such as placental fragmentation, subchorionic and intraparenchymal hemorrhage, areas of infarction and acute chorioamnionitis.
